# A Hybrid Minimally Invasive Atrial Fibrillation Ablation Procedure Using Unilateral Thoracoscopy and Endocardial Pulsed Field Ablation: An Early Feasibility Study

**DOI:** 10.3390/jcdd12040145

**Published:** 2025-04-09

**Authors:** Ivan Eltsov, Luigi Pannone, Domenico Giovanni Della Rocca, Massimiliano Marini, Giacomo Talevi, Andrea Maria Paparella, Pasquale Vergara, Erwin Ströker, Juan Sieira, Gian-Battista Chierchia, Carlo de Asmundis, Mark La Meir

**Affiliations:** 1 Heart Rhythm Management Centre, Universitair Ziekenhuis Brussel—Vrije Universiteit Brussel, European Reference Networks Guard-Heart, 1090 Brussels, Belgium; ivan.eltsov@vub.be (I.E.); luigi.pannone@uzbrussel.be (L.P.); domenico.dellarocca@uzbrussel.be (D.G.D.R.); giacomo.talevi@uzbrussel.be (G.T.); andrea.paparella@uzbrussel.be (A.M.P.); pasqualevergara@hotmail.com (P.V.); erwin.stroker@uzbrussel.be (E.S.); juan.sieira@uzbrussel.be (J.S.); jeanbaptiste.chierchia@uzbrussel.be (G.-B.C.); 2 Cardiac Surgery Department, Universitair Ziekenhuis Brussel—Vrije Universiteit Brussel, 1090 Brussels, Belgium; mark.lameir@uzbrussel.be; 3 Department of Cardiology, S. Chiara Hospital, 38122 Trento, Italy; massimiliano.marini@apss.tn.it

**Keywords:** hybrid epicardial ablation, video-assisted thoracoscopy, pulsed field ablation, 3D mapping

## Abstract

(1) Objective: To examine the efficiency and efficacy of using endovascular mapping and pulsed field ablation in the setting of a hybrid video-assisted thoracoscopic atrial fibrillation (AF) ablation procedure. (2) Methods: Eleven consecutive patients underwent hybrid video-assisted thoracoscopic epicardial ablation and left atrial appendage exclusion followed by endocardial ablation using pulsed field ablation energy. The completeness of epicardial and endocardial lesion sets were assessed using 3D electro-anatomical mapping. (3) Results: Left atrial appendage (LAA) exclusion and durable pulmonary vein isolation (PVI) and posterior wall isolation (PWI) were achieved in all patients. The endovascular part of the necessary lesion set using PFA energy was successful in 100% of the patients. All patients remained in SR during the 12-month follow-up period. (4) Conclusions: Our study confirms the feasibility of using endovascular pulsed field ablation to complete previously performed epicardial lesion sets during the hybrid AF ablation procedures, without extending the procedure time or increasing the risk of complications.

## 1. Introduction

Atrial fibrillation (AF) is the most common sustained arrhythmia, and its prevalence is expected to double by 2030 [1,2]. Despite numerous technological advancements [3,4], the gold-standard surgical treatment for AF is the Cox-Maze IV procedure (CMP), which has proven to be safe and effective [5,6].

Despite the fact that the guidelines set out by the Society of Thoracic Surgeons and European Association for Cardio-Thoracic Surgery (EACTS) and the European Society of Cardiology (ESC) have strongly recommended the concomitant AF ablation procedure [7,8,9] since 2017, its adoption during concomitant cardiac surgery remains low, at only 22% [10].

These recommendations can be applied to concomitant open-chest procedures, but performing the standalone open-chest epicardial AF ablation procedure for many clinicians seems more invasive compared to endovascular ablations, and this remains a major limitation factor of this technique. Therefore, other treatments (mainly antiarrhythmic drug therapy and catheter ablation) preferred as the first choice.

Recently, different research groups have developed various hybrid minimally invasive techniques, combining the effectiveness of surgical procedures with the safety profile of endovascular catheter ablation techniques [11,12,13,14].

These procedures showed promising results; however, there are certain intrinsic limitations. In particular, the surgical stage might be limited by extreme variations in the patient anatomy, where it becomes impossible to reach certain anatomical structures using video-assisted thoracoscopy (pericardial adhesions, obesity, excess fat tissue, anatomical variances), while endovascular ablation techniques (especially radiofrequency) do not often provide the transmurality required to complete the lesion set.

A new energy ablation source, called pulsed-electric field ablation (PFA), has recently shown promising data for catheter ablation. Its efficacy has been comparable with that of standard catheter RFA and cryoablation, with significantly shorter procedural times and lower complication rates reported [15,16,17].

Our centre was among early users of this ablation modality, and in this study, we aim at analyzing the efficacy and safety of PFA in the setting of previously developed hybrid ablation techniques for atrial fibrillation [18,19].

## 2. Materials and Methods

This is a first-experience single-center retrospective feasibility study including 11 consecutive patients undergoing hybrid video-assisted thoracoscopic (VATS) AF ablation with endovascular PFA lesion sets at our hospital. The inclusion criteria were as follows: (1) persistent AF with hybrid unilateral thoracoscopic ablation procedure and left atrial appendage closure, and (2) the use of a PFA energy source during the endovascular stage. The study complies with the Declaration of Helsinki and was approved by local ethics committees; informed consent was obtained from the subjects before inclusion in the study. All data were collected and updated in the registry of the Universitair Ziekenhuis, Brussels, and approved by the institutional ethics committee.

The hybrid AF ablation was performed as a two-step procedure: (I) thoracoscopic ablation followed by (II) endocardial mapping and eventual ablation. The procedure was performed in the electrophysiology laboratory as previously described [19].

**Figure 1:**
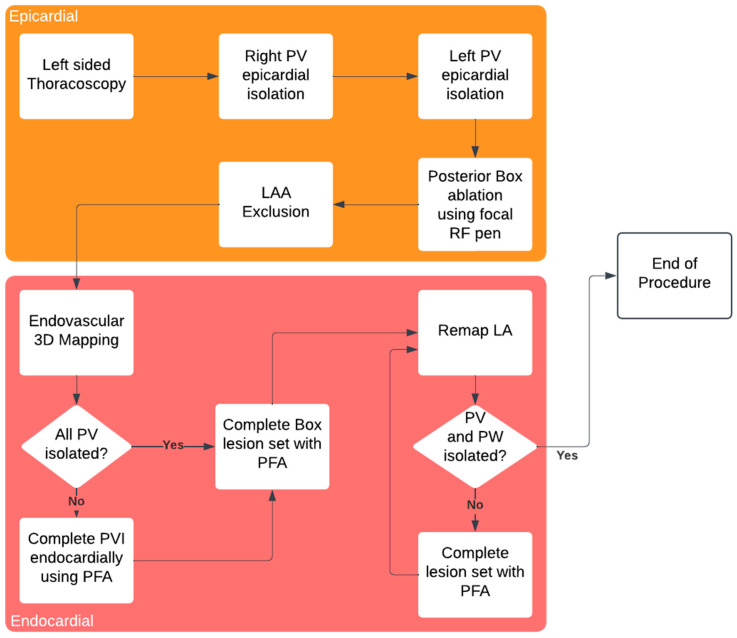
Hybryd AF ablation procedure workflow.

The procedure workflow is summarized in Figure 1.

The following data were collected: clinical history including demographic, race/ethnicity and biometric data; antiarrhythmic drugs (AADs); procedural characteristics; epicardial lesion set assessment prior to the endocardial stage, endocardial lesion set and the final procedure results.

### 2.1. Devices

**Figure 2:**
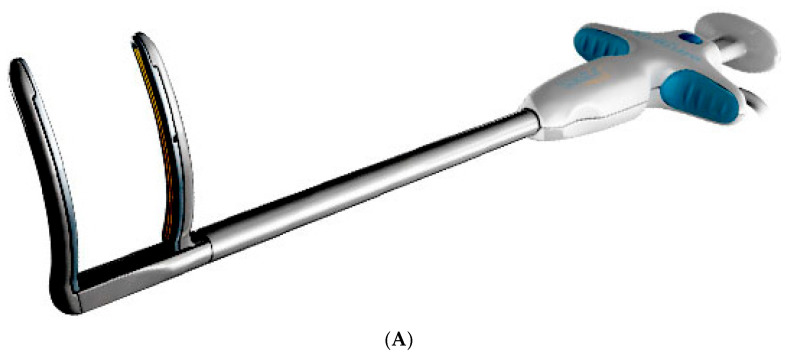

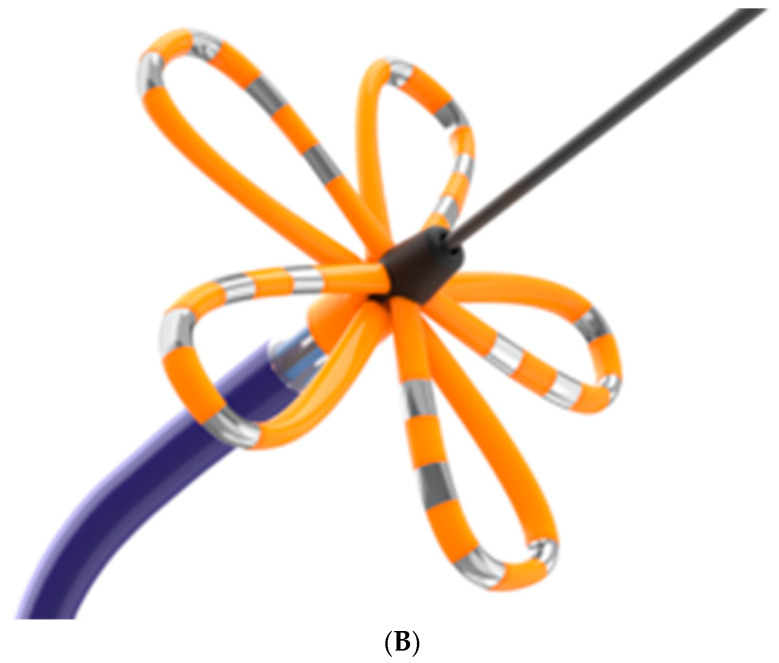
Devices Used During the procedure. (**A**) Atricure RF clamp. (**B**) FaraWave PFA catheter.

Epicardial VATS ablation of pulmonary veins was performed using the Isolator Synergy bipolar RF clamp (Atricure, Mason, OH, USA); the linear RF probe CoolRail (Atricure, Mason, OH, USA) was used for ablation of the posterior wall of the left atrium and mitral isthmus (Figure 2A). Endocardial PFA was performed using the FaraWave Catheter (Boston Scientific, Malborough, MA, USA), Figure 2B.

### 2.2. Surgical Procedure

**Figure 3:**
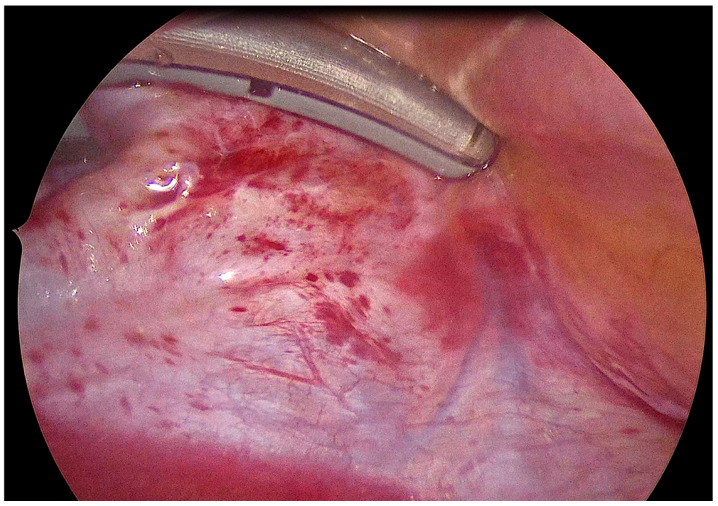
Epicardial isolation of left pulmonary veins using the bipolar RF clamp.

General anesthesia was administered with a double-lumen endobronchial tube for selective lung ventilation. In all patients, unilateral thoracoscopic access with 5 mm ports was used. As previously described [18], antral isolation of both pairs of PVs was performed across four to six applications using a bipolar radiofrequency (RF) clamp, and was introduced using a LumiTip (Atricure, Mason, OH, USA) with the 5 mm port removed (Figure 3). PVI was assessed during the endocardial stage by mapping and pacing through an endovascular catheter (exit block). Left atrial posterior wall isolation (LAPWI) was performed epicardially in all patients with two lines: (i) a roof line (connecting both superior PVs) and (ii) an inferior line (connecting both inferior PVs). Either a bipolar RF pen or a linear pen device (Isolator Pen and Coolrail, Atricure, Mason, OH, USA) was used for LAPWI. The posterior mitral line, connecting the mitral annulus to the left inferior PV, usually performed endocardially, while the ablation of the Ligament of Marshall can be performed epicardially, if deemed appropriate. Left atrial appendage (LAA) clipping was performed in 10 patients (90.9%) with the AtriClip device (AtriCure, Mason, OH, USA).

### 2.3. Endovascular Procedure

**Figure 4:**
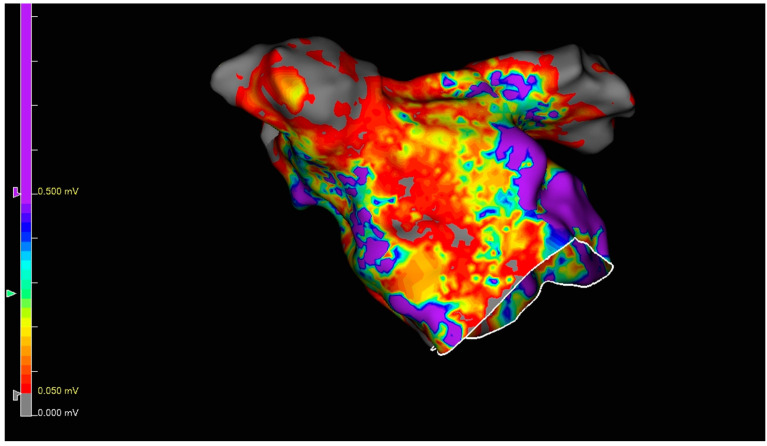
Endocardial voltage map after complete epicardial ablation lesion set. The purple color shows a high voltage (not ablated).

Percutaneous endocardial mapping and ablation was performed via the femoral venous approach. A decapolar catheter (Biotronik, Berlin, Germany, or Abbott, Abbott Park, IL, USA) was placed in the coronary sinus under fluoroscopy, and a single transseptal puncture was performed with a long sheath (SL0, Abbott, USA) and BRK needle under TOE and fluoroscopy guidance. Before the transseptal puncture, full heparinization (1000 U heparin per 10 kg body weight) was given with a targeted activated clotting time of 300 s. An electroanatomic map of the left atrium (LA) was performed either with EnsiteX system (Abbott) using the multipolar grid-mapping catheter (Advisor HD Grid, Abbott, Abbott Park, IL, USA) or with a basket-shaped mapping catheter (IntellaNav Orion, Boston Scientific, Malborough, MA, USA) and Rhythmia system (Boston Scientific, Malborough, MA, USA). PVI and LAPWI were assessed by voltage map (Figure 4) and pacing maneuvers (entrance and exit block). The voltage map was analyzed afterwards to identify possible gaps in PVI and PW. Then, the SL0 sheath was exchanged with the steerable (15F) long sheath (FaraDrive, Boston Scientific, Malborough, MA, USA), and the ablation PFA catheter was introduced into the LA.

PVI and LAPWI were performed with a 31 mm basket/flower-shaped PFA ablation catheter. Additional lesions were placed endocardially based on clinical indication and the physician’s judgement. Possible, but not mandatory, additional lesions included the following: the anterior mitral line connecting the mitral annulus to the right superior PV; the posterior mitral line connecting the mitral annulus to the left inferior PV; and left and right continuous fractionated atrial electrogram (CFAE) mapping and ablation. In patients who did not convert to sinus rhythm during the ablation, electrical cardioversion was performed. The bidirectional block was confirmed for all ablation lines with pacing maneuvers, and final remapping was performed using the same mapping catheter as at the beginning of the procedure.

After the procedure, all patients underwent continuous telemetric monitoring until discharge from the hospital. Before discharge, TTE was performed in all patients to exclude post-procedural pericardial effusion. LMWH was started the same evening following the ablation, and on the third postoperative day, OAC was reinitiated. Patients were restarted with previous AADs within one week after ablation. Oral anticoagulation and AADs were continued for at least 3 months. AADs were maintained or stopped following current guidelines after 3 months, based on clinical judgement and rhythm at follow-up visit.

### 2.4. Follow-Up

All patients were followed-up for at least 12 months after the procedure, including Holter monitoring at 6 and 12 months after the procedure (in addition to Holter monitoring at 3 months).

### 2.5. Statistical Analysis

The analysis was performed using R software version 3.6.2 (R Foundation for Statistical Computing, Vienna, Austria). All variables were tested for normality with the Shapiro–Wilk test. Normally distributed variables were described as mean ± standard deviation, and groups were compared through ANOVA, either as a paired or unpaired *t*-test as appropriate. Non-normally distributed variables were described as median (interquartile range) and compared using the Mann–Whitney or Wilcoxon signed-rank test, as appropriate. The categorical variables were described as frequencies (percentages) and compared by the χ^2^ or Fisher’s exact test, as appropriate. A *p*-value < 0.05 was considered statistically significant.

## 3. Results

### 3.1. Study Population Characteristics

Eleven consecutive patients who underwent Hybrid VATS and endocardial PFA procedure met the inclusion criteria and were analyzed. A subgroup of patients with anatomical difficulties which led to incomplete epicardial lesion sets was identified. In two patients, the right-sided veins were not reachable via left-sided thoracoscopy due to extreme anatomical difficulties—they had severe adhesions from previous interventions or/and pericarditis, which made it dangerous to access sinus transversus and sinus obliquus. In these same two patients, PWI was also not performed. Two patients had extreme amounts of fat on the posterior aspect of the LA, which made it impossible to complete PWI, and one patient had excessive adhesions preventing access to the sinus transversus; therefore, RPV was not ablated, but as the sinus obliquus was accessible, the posterior wall lesion set was partially delivered.

Patient characteristics are summarized in Table 1.

### 3.2. Intraprocedural Assessment of Epicardial Lesion Set

At the surgical stage, the epicardial lesion set was not completed in four patients. The remapping after the epicardial lesion set and before the endovascular stage of the procedure showed the following results: (1) For left-sided pulmonary veins (LPV) complete isolation was confirmed in 100% of patients, whereas for right-sided veins (RPV), when epicardial PVI was possible, remapping showed a 100% isolation rate. In three patients, however, RPV isolation was not performed due to anatomical difficulties, and these three PVs were surprisingly not found to be isolated at initial endocardial mapping.

Gaps in LAPWI were identified in four patients (Figure 4). Assessment data for the intraprocedural epicardial lesions are summarized in Table 2.

The endocardial lesion set has been unified, so PVI and PWI were performed in 100% of patients; in three patients, a mitral line was performed. The PFA lesion set has been summarized in Table 3.

### 3.3. Acute Procedure Results

At the end of the ablation procedure, an endocardial map was performed (Figure 4); the mapping outcomes and other procedural characteristics are summarized in Table 4. Unsurprisingly, the duration of surgical part, the total procedure duration, and the number of extra applications were higher in the group with an incomplete epicardial lesion set.

### 3.4. Safety

No major periprocedural adverse events were observed in any patients.

### 3.5. Follow-Up Results

All patients were followed-up for at least 12 months after the procedure, and neither arrhythmia reoccurrences nor recurrent or extended hospitalisations were observed.

## 4. Discussion

Pulsed field ablation (PFA) has recently emerged in the field of electrophysiology, offering promising safe and efficient results. As previously described, hybrid AF ablation procedures using unilateral approach are associated with good long-term outcomes [18]. On the other side, the use of PFA energy is associated with significantly shorter procedure times and a lower vein reconnection rate (compared to AAD and other energy sources) with a superior safety record [20,21]. However, its superiority over epicardial surgical ablation remains debatable, especially when left atrial appendage exclusion is also indicated. For these patients, a minimally invasive surgical alternative might remain the procedure of choice. Furthermore, despite the excellent long-term results of epicardial surgical procedures, there is a small cohort of patients for whom a complete lesion set is impossible when using the unilateral VATS approach due to anatomical difficulties (pleural spikes, excessive amount of epicardial fat, technical difficulties). In this study, we assessed the feasibility of using novel energy sources and ablation catheters in combination with HVATS for AF ablation to complete epicardial lesion sets. The results of our study show that the endocardial ablation of LA following the epicardial stage can be very effective and can help to complete the left atrial lesion set in challenging procedures while remaining minimally invasive.

As with any other hybrid VATS procedure [22], the current approach with PFA confirms the importance of 3D anatomical mapping after epicardial and endocardial ablation. Three-dimensional EAM allows us to verify the transmurality of epicardial lesions and target all potential gaps to ensure the completeness of substrate modification.

As described by other groups, PFA has an important disadvantage—the temporary stunning effect around the application site which can lead to misinterpretation of isolation confirmation. Therefore, the optimal workflow in these procedures should include performing VATS LAAC and epicardial ablation before mapping and endovascular ablation, as suggested in Figure 1.

Finally, using PFA allows us to avoid important procedural complications due to its excellent safety record [23,24], which makes it ideal for use in completing lesion sets during hybrid AF procedures.

## 5. Conclusions

Our study confirms the feasibility of using PFA during hybrid VATS ablation of atrial fibrillation with concomitant LAA exclusion. It also highlights the importance of electroanatomical mapping while performing these procedures. This approach allows us to achieve durable lesions and prevent arrhythmia recurrence in patients where a full epicardial lesion set is impossible. Further studies are necessary to compare the efficiency of such an approach with classic minimally invasive surgical AF ablation procedures.

## 6. Limitations

The patient cohort is very limited due to limited indications and early experience; therefore, this study has to be considered as a feasibility study only. Follow-up is limited. All procedures were performed by a single experienced surgeon in a referral center who is an expert at VATS hybrid ablation.

## Figures and Tables

**Table 1:** Patient characteristics.

	Total (*N* = 11)	Normal Epicardial Stage (*N* = 7)	Anatomical Difficulties Incomplete VATS Stage (*N* = 4)	*p*-Value
Age (years)	65.3 ± 9.3	64.6 ± 10.3	70.3 ± 9.2	0.81
Sex (male)	10 (90%)	6 (85.7%)	4 (100%)	0.9
BMI (Kg/m^2^)	29.6 ± 4.2	28.7 ± 3.8	31.3 ± 5.1	0.56
Cordarone	3	0 (0%)	2 (50%)	0.048
Beta Blockers	4	3 (42.8%)	1 (25%)	0.1
IC AAD	3	2 (28.6%)	1 (25%)	0.9
Index Procedure	0 (0%)	0 (0%)	0 (0%)	N/A
Number ReDO	2.8	3 (42.8%)	2.75 (68.7%)	0.71
Epicardial first	11 (100%)	7 (100%)	4 (100%)	1.0

N/A—not available.

**Table 2:** Results of initial mapping after epicardial ablation.

	Total (*N* = 11)	Epicardial Ablation Complete (*N* = 7)	Anatomical Difficulties Incomplete VATS Stage (*N* = 4)	*p*-Value
LPV performed	11 (100%)	7 (100%)	4 (100%)	N/A
Nr of LPV epi applications	4.2 ± 0.7	4 ± 1.0	4 ± 0.0	0.292
RPV performed	8 (72.7%)	7 (100%)	1 (25%)	N/A
Nr of RPV epi applications	4.6 ± 0.7	4.8 ± 0	4 ± 1.0	0.008
PW epi performed	8 (72.7%)	7 (100%)	1 (25%)	N/A
Nr of PW epi applications	12.8 ± 8.0	11.8 ± 1.1	24 ± 8.0	0.31
LAAC performed	9 (81.8%)	7 (100%)	4 (100%)	1
LSPV isolated	11 (100%)	7 (100%)	1 (25%)	1
LIPV isolated	11 (100%)	7 (100%)	1 (25%)	1
RSPV isolated	8 (72.7%)	7 (100%)	3 (75%)	0.87
RIPV isolated	8 (72.7%)	7 (100%)	3 (75%)	0.87
PW isolated	5 (40%)	6 (85.7%)	3 (75%)	0.8

N/A—not available.

**Table 3:** Endocardial PFA lesion summary.

	Total (*N* = 11)	Epicardial Ablation Complete (*N* = 7)	Anatomical Difficulties Incomplete VATS Stage (*N* = 4)	*p*-Value
Nr FP applications LSPV	11 ± 3.1	10.5 ± 3.4	12.5 ± 3.0	0.69
Nr FP applications LIPV	10.6 ± 3.6	10.6 ± 2.8	10.7 ± 4.9	0.95
Nr FP applications RSPV	10.2 ± 3.7	10.8 ± 2.7	11.2 ± 5.3	0.87
Nr FP applications RIPV	11 ± 2.5	10 ± 2.5	10.4 ± 4.9	0.83
Nr FP applications PW	39.7 ± 16.5	37.8 ± 20.2	42.25 ± 13.1	0.72
Nr FP applications MI	21 ± 12.4	11 ± 12.7	10 ± 13.9	0.95

**Table 4:** Procedural characteristics and efficacy.

	Total (*N* = 11)	Epicardial Ablation Complete (*N* = 7)	Anatomical Difficulties Incomplete VATS Stage (*N* = 4)	*p*-Value
Procedure duration	206 ± 46.3	217 ± 52.7	193 ± 39.6	0.458
Surgery duration	55 ± 21.2	65.6 ± 17.7	42.5 ± 19.7	0.106
Endo duration	61.3 ± 29.0	62.2 ± 22.9	60.2 ± 35.8	0.928
Fluoro time	23.8 ± 6.5	26.9 ± 7.1	19.9 ± 2.8	0.109
Dosage	157.5 ± 77.0	181.4 ± 94.9	127.5 ± 40.7	0.329

## Data Availability

Study data are available upon request.

## Abbreviations

The following abbreviations are used in this manuscript:

AF	Atrial Fibrillation
PFA	Pulsed Field Ablation
EAM	Electro Pulsed Field Anatomical Mapping
RF	Radio Frequency
VATS	Video-Assisted Thoracoscopy
CMP	Cox-Maze Procedure
LPV	Left Pulmonary Veins
LSPV	Left Superior Pulmonary Vein
LIPV	Left Inferior Pulmonary Vein
RPV	Right Pulmonary Veins
RSPV	Right Superior Pulmonary Vein
RIPV	Right Inferior Pulmonary Vein
LAA	Left Atrial Appendage
LAAC	Left Atrial Appendage Occlusion
LAPW	Left Atrial Posterior Wall
PML	Posterior Mitral Line
